# Damage-induced pyroptosis drives endogenous thymic regeneration by activating the purinergic receptor P2Y2

**DOI:** 10.1038/s41419-025-08345-x

**Published:** 2026-01-03

**Authors:** Sinéad Kinsella, Cindy A. Evandy, Kirsten Cooper, Erin Kirsche, Makya Warren, Paul deRoos, Antonella Cardinale, Lorenzo Iovino, David Granadier, Colton W. Smith, Kayla Hopwo, Lucas B. Sullivan, Enrico Velardi, Jarrod A. Dudakov

**Affiliations:** 1 Division of Translational Science and Therapeutics, Program in Immunology, Fred Hutchinson Cancer Center, Seattle, WA USA; 2 Immunotherapy Integrated Research Center, Fred Hutchinson Cancer Research Center, Seattle, WA USA; 3 Department of Pediatric Hematology and Oncology, Bambino Gesù Children’s Hospital, IRCCS, Rome, Italy; 4 Medical Scientist Training Program, University of Washington, Seattle, WA USA; 5 Human Biology Division, Fred Hutchinson Cancer Center, Seattle, WA USA; 6 Department of Immunology, University of Washington, Seattle, WA USA

**Keywords:** Cell death and immune response, Haematopoiesis

## Abstract

T cell recovery is critical following damage, such as hematopoietic cell transplantation (HCT), with increased reconstitution associated with improved clinical outcomes. Endogenous thymic regeneration, a crucial process for restoring immune competence following cytoreductive therapies such as HCT conditioning, is often delayed, limiting T cell reconstitution. Fully understanding the molecular mechanisms driving regeneration is therefore crucial for uncovering therapeutic targets that can be exploited to enhance thymic function. Here, we identified that CD4+ CD8+ thymocytes rapidly and acutely undergo lytic cell death, specifically pyroptosis, following acute damage caused by ionizing radiation, and release damage-associated molecular patterns (DAMPS) into the thymic microenvironment, including ATP. Extracellular ATP stimulates the P2Y2 purinergic receptor on thymic epithelial cells (TECs)—a stromal cell crucial for supporting T cell development—resulting in the upregulation *FOXN1*, the master TEC transcription factor. Targeting the P2Y2 receptor with a P2Y2 agonist, UTPγS, promotes rapid regeneration of the TEC compartment in vivo following acute damage. These findings reveal a novel damage-sensing mechanism employed by the thymus where thymocytes adopt an alternative cell death mechanism which promotes thymic repair via P2Y2 signaling in TECs. This work identifies P2Y2 as a promising therapeutic target for enhancing thymus regeneration and improving immune recovery after HCT.

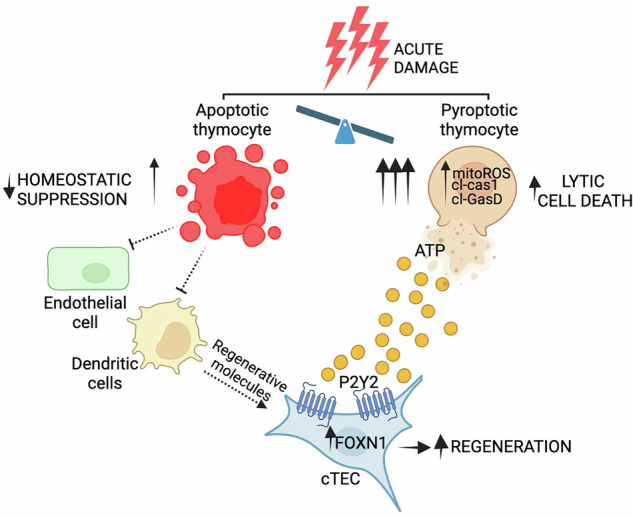

## Introduction

Competent T cell development relies on the efficient functioning of the thymus, which is extremely sensitive to acute insults, such as that caused by cytoreductive therapies [1]. This presents the important clinical problem of opportunistic infections and malignant relapse during periods of extended lymphopenia, including in the setting of hematopoietic cell transplantation [2, 3]. Moreover, thymic function progressively declines with age, resulting in reduced export of newly generated naïve T cells and reduced responsiveness to new antigens and vaccines [4–6]. The thymus has a remarkable ability to endogenously regenerate [7–9], however, age-related deterioration drastically erodes this regenerative capacity [10]. Harnessing this intrinsic reparative response has the potential to expedite reconstitution of naïve T cells and improve immune function, in the settings of both age and acute cytoreductive damage. However, much remains unknown about the molecular regulators that govern this critical process.

We have previously identified that IL-22 and BMP4 represent two distinct pathways that facilitate endogenous repair in the thymus and, in the case of BMP4, is largely mediated by induction in the expression of *Foxn1* [8, 11]. FOXN1 is the essential thymic epithelial cell (TEC) transcription factor; not only crucial for the generation and function of TECs - the major stromal cell compartment of the thymus - but also for TEC maintenance with declining expression associated with age-related thymic involution [12]. We have previously identified that the constitutively high levels of homeostatic apoptosis in the steady state thymus, which governs negative selection events, suppress the production of BMP4 and IL-23 (an upstream regulator of IL-22) [13]. Following injury, the depletion of apoptotic thymocytes relieves this suppression, promoting the production of these regenerative factors and facilitating thymic repair [8, 11, 13], and the depletion of apoptotic thymocytes after injury promotes their production leading to regeneration [11].

Cell death is a sophisticated and tightly controlled, intricate event, with the mitochondria playing a central role in regulating this process [14–17]. Given the robust depletion of thymocytes after acute damage preceding the activation of these reparative pathways, we hypothesized that in addition to the decrease in apoptosis-mediated suppression of regeneration we have previously observed [13], an alternative form of cell death may simultaneously occur, providing a mechanism for the triggering of tissue regeneration. Notably, necrotic cell death has been tightly linked to tissue regeneration across multiple organs, and we have recently found that release of pro-inflammatory IL-18 after injury is limiting of thymic repair [18–23, 24]. We investigated the effects of acute damage on lytic cell death in the thymus and identified that mitochondrial dysregulation precedes the caspase 1 activation, a key event in the initiation of pyroptotic cell death. We further identified the release of DAMPs, including ATP, into the thymic microenvironment. Extracellular ATP activates the purinergic receptor P2Y2, a G-coupled receptor that has been previously demonstrated to regulate regenerative pathways in hepatocytes [25] and neurons [26] by triggering intracellular Ca2+ cascades.

Here, we describe a novel and central role for P2Y2 activation in thymic repair. These findings identify a previously unrecognized function of pyroptotic cell death in the regulation of T cell development and thymic repair and provide a targetable therapeutic strategy to enhance immune function.

## Results

### CD4+ CD8+ thymocytes undergo robust but transient increase in pyroptosis after damage

**Fig. 1:**
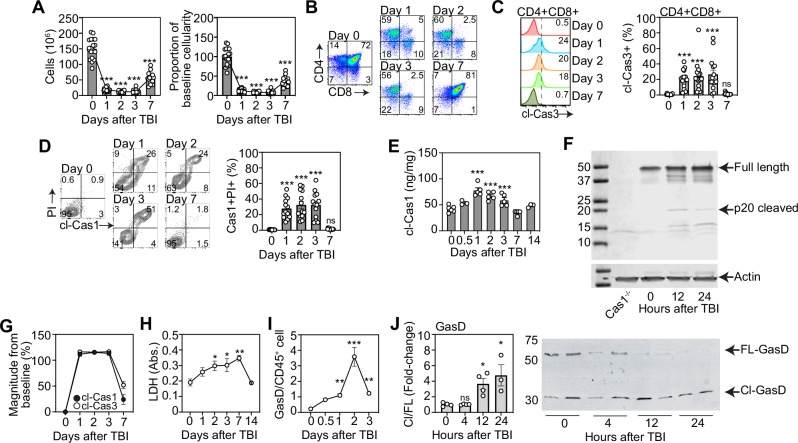
An acute increase in pyroptotic DP thymocytes triggers thymus regeneration after damage. **A**–**E** The thymus from 6 to 8 week-old C57/BL/6 mice was analyzed at days 0, 1, 2, 3, and 7 following sublethal total body irradiation (TBI, 550 cGy). **A** Total thymic cellularity and proportion of cellularity as a function of baseline cellularity (n = 15–19/timepoint from 5 independent experiments); **B** Concatenated flow cytometry plot of CD4 vs CD8 (gated on viable CD45+ cells) (n = 9–13 from 3 to 4 independent experiments); **C** Concatenated flow cytometry histogram from a representative experiment showing cleaved caspase-3 in DP thymocytes (Gated on CD45+ CD4+ CD8+ cells) and bar graph showing proportion of cleaved-cas3+ DP thymocytes (n = 15–16/timepoint from 5 independent experiments); **D** Concatenated flow cytometry plot from a representative experiment showing cleaved-caspase1 and PI in DP thymocytes (gated on CD45+ CD4+ CD8+ cells) and bar graph with proportions (n = 13/timepoint from 4 independent experiments); **E** Cleaved caspase 1 levels in DP thymocytes measured by ELISA at 0, 12 h, 1, 2, 3, 7 and 14 days after TBI (n = 3–6 mice/group) **F** Western Blot analysis of thymocytes at 0, 12 and 24 h after TBI showing full length and cleaved caspase 1 levels (n = 3 thymi/group); **G** Magnitude of change in expression of cleaved-caspase-1 and cleaved-caspase-3 in DP thymocytes after TBI (n = 10–15/timepoint/condition from 4–5 independent experiments); **H** Lactate dehydrogenase levels were measured in the thymus supernatant of mice (n = 4 mice/group); **I** Gasdermin D levels were measured in CD45+ cells from the thymus at days 0, 1 2, 3, 7, and 14 post TBI (n = 3–4 mice/group from 2 to 3 independent experiments). **J** Thymuses form steady state and TBI-treated mice were harvested 4, 12, and 24 h following TBI and prepared for western blot. Full-length and cleaved gasdermin D levels were measured by western blot (n = 4 mice/condition, unpaired two-tailed t-test). Summary data represent mean ± SEM. *, p < 0.05; **, p < 0.01; ***, p < 0.001.

Most thymocytes—particularly CD4+ CD8+ double positive (DP) thymocytes—undergo apoptosis as a function of the selection processes fundamental for T cell development [27–29]. We previously identified that homeostatic apoptotic events suppress the production of multiple regenerative molecules in the thymus by promoting activation of TAM receptors, which sense phosphatidylserine (PtdSer), bridging apoptotic thymocytes to surrounding stromal cells [13]. Although steady-state thymocyte apoptosis attenuates the regenerative response, acute damage results in a marked depletion of cellularity (Fig. 1A), indicating a substantial increase in cell death and suggesting that alternate non-apoptotic forms of cell death may be acutely induced after injury and prior to the induction of regenerative molecules. DP thymocytes are the most abundant thymic population, comprising ~80% of a thymus at baseline, and are highly sensitive to damage, such as after sub-lethal total body irradiation (TBI, 550 cGy) (Fig. 1B). Given this sensitivity, we examined the effects of acute damage on cell death mechanisms within the thymocytes compartment. Consistent with previous findings, we observed considerable cleavage of caspase-3 (a key apoptosis executioner caspase) in thymocytes (Fig. 1C), demonstrating their sensitivity to damage [28, 30]. However, we further identified significant cleavage of caspase-1 in dying thymocytes, suggesting that in addition to immunologically silent apoptosis, pyroptosis may transiently be induced following injury (Fig. 1D). We further identified increased levels of cleaved-caspase-1 (cl-caspase-1) by ELISA and Western Blot analysis (Fig. 1E, F). Moreover, direct comparison revealed a similar magnitude of activation of both caspase-3 and caspase-1 after damage in DP thymocytes (Fig. 1G). Consistent with this induction of caspase 1 cleavage, we determined that both extracellular lactate dehydrogenase (LDH) (Fig. 1H) and cleaved gasdermin D (Fig. 1I, J) were increased in the thymus after TBI, indicating a simultaneous induction of pyroptosis following acute damage.

### Damage-induced mitochondrial dysregulation sensitizes DP thymocytes for pyroptosis

**Fig. 2:**
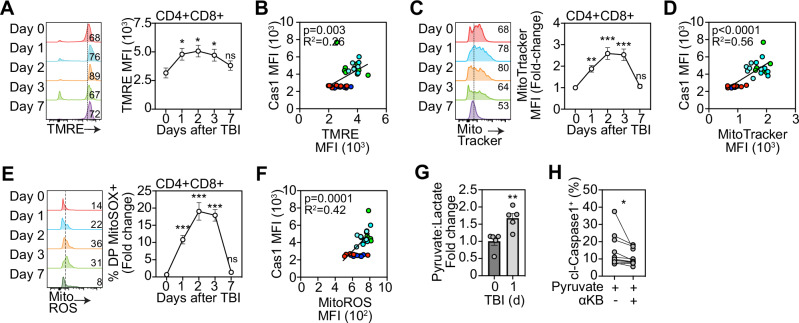
Mitochondrial dysregulation positions DP thymocytes for pyroptosis. **A**–**F** Mitochondrial function was analyzed in the thymus isolated from 6-8-week-old C57/BL/6 mice at days 0, 1, 2, 3, and 7 following sublethal total body irradiation (TBI, 550 cGy). **A** Mitochondrial membrane potential assessed by staining of TMRE. Concatenated histogram of TMRE on DP thymocytes (left), quantification of TMRE+ proportions (right) (n = 8–12 mice from 3 separate experiments); **B** Correlation of TMRE MFI with Caspase-1 MFI (n = 3–8 from 3 independent experiments); **C** Mitochondrial mass assessed by MitoTracker Green. Concatenated histogram of TMRE on DP thymocytes (left), quantification of TMRE+ proportions (right) (n = 9–10 mice from 3 independent experiments); **D** Correlation of MitoTRACKER+ thymocytes with Caspase-1 MFI (n = 6/timepoint from 3 independent experiments); **E** Mitochondrial ROS was assessed by staining for MitoSOX. Concatenated histogram of TMRE on DP thymocytes (left), quantification of TMRE+ proportions (right) (n = 5–7 mice from 2 separate experiments); **F** Correlation of MitoROS levels with Caspase-1 MFI (n = 5–7/timepoint from 2 independent experiments); **G** Intracellular lactate and puyruvate levels were measured in freshly isolated thymocytes from untreated and TBI-treated mice and represented as a ratio of pyruvate:lactate (n = 5 mice/group from 2 separate experiments); **H** Freshly isolated thymocytes from untreated mice were incubated with RPMI supplemented with pyruvate (5 mM) for 4 h plus a-ketobutyrate (200 µM). cl-caspase 1 levels were measured using flow cytometry (n = 13–14 mice from 4 separate experiments). Summary data represent mean ± SEM. *, p < 0.05; **, p < 0.01; ***, p < 0.001.

In addition to its crucial role in cellular metabolism, the mitochondria is a central gatekeeper of cell death [14, 15, 31, 32]. As thymocytes undergo high levels of homeostatic cell death and have a high level of metabolic plasticity [33–35], we examined whether damage-induced metabolic adaptations influence cell death pathways. Firstly, we measured mitochondrial membrane potential using TMRE and revealed a marked acute induction of mitochondrial membrane hyperpolarization (Fig. 2A), correlating with increased cl-caspase 1 levels (Fig. 2B). Further evidence of a damage-induced dysregulated metabolic phenotype was observed in thymocytes with an increase in mitochondrial mass, suggesting an acute impairment of mitophagy in DPs after acute damage (Fig. 2C), which also positively correlated with Cas1 cleavage (Fig. 2D). The acute increases of both mitochondrial membrane potential and mitochondrial mass were resolved by day 7 following damage, coinciding with the pattern we observed with caspase 1 cleavage and the re-establishment of the physiological apoptosis:pyroptosis balance (Fig. 1G).

Assembly and activation of the NLRP3 inflammasome is a key event leading to caspase 1 cleavage [36–38], and mitochondrial ROS promotes this cascade [39–42]. We therefore hypothesized that enhanced mitochondrial activation in DPs led to increased production of mitochondrial ROS (mitoROS) priming DPs for pyroptosis. We identified a rapid transient elevation in mitoROS levels after damage that correlated with caspase-1 cleavage (Fig. 2E, F). Glutathione is an antioxidant thiol that responds to increased ROS levels [43], and imbalance between glutathione and ROS can lead to cellular instability and death [44]. Consistent with this pathway, we observed an acute increase in glutathione levels in DPs, similarly resolved within 7 days following damage (Supplementary Fig. 1A). Next, to further characterize damage-induced metabolic alterations in DP thymocytes, we measured the pyruvate-to-lactate ratio and observed a significant shift towards increased pyruvate levels early after injury (Fig. 2G). To assess whether this metabolic shift contributes to pyroptosis in DP thymocytes we treated thymocytes with α-ketobutyrate (α-KB), a competitive inhibitor of the pyruvate converting enzyme PDH that decreases the conversion to acetyl-CoA [45, 46], and identified a reduction in cleaved caspase-1 levels, suggesting that alterations in mitochondrial metabolism contribute to caspase 1 cleavage in thymocytes (Fig. 2H).

### Extracellular ATP induces Foxn1 expression in cTECs

**Fig. 3:**
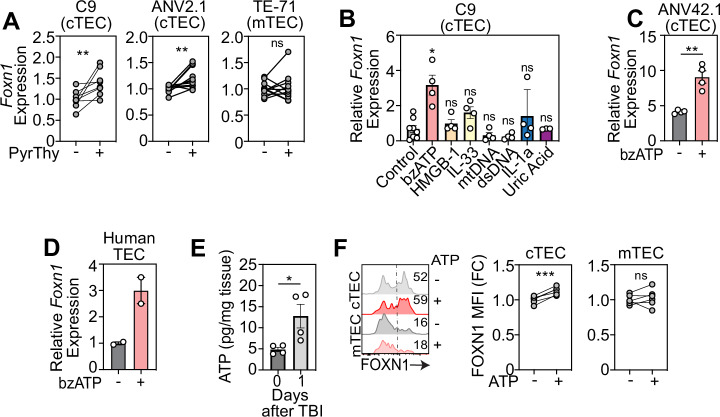
Extracellular ATP induces upregulation of FOXN1 in cTECs. **A** TEC cell lines were co-cultured with freshly isolated thymocytes treated to induce or inhibit pyroptosis and *Foxn1* expression was measured by qPCR 20 h after co-culture, in C9s (n = 8–11 thymuses from 3 separate experiments), ANV42.1 (n = 8 thymuses from 3 separate experiments), and TE-71 (n = 11 from 3 separate experiments); **B** The cTEC cell line C9 was treated with a panel of DAMPs and *Foxn1* transcription was measured in by qPCR 20 h following incubation (n = 3–4 separate experiments); **C** A second cTEC cell line (ANV42.1) was treated with ATP (100 µM) and *Foxn1* expression was measured after 20 h (n = 3 separate experiments); **D** Freshly isolated human cTECs were treated with ATP (100 µM) and *Foxn1* expression was measured after 20 h (n = 2); **E** ATP levels measured by colorometric assay at day 0 and 1 after TBI (550 cGy) (n = 4/timepoint); **F** Steady-state thymuses were harvested from 8-week-old C57BL/6 J mice and enzymatically digested. The cells were plated at 2 × 10^6^ cells/well and stimulated with 100 µM bzATP. FOXN1 expression was assessed by flow cytometry 20 h following bzATP treatment (n = 5–6 mice from 2 independent experiments). Summary data represent mean ± SEM. *, p < 0.05; **, p < 0.01; ***, p < 0.001.

Building on our findings that lytic cell death of DPs may promote regeneration, we investigated whether pyroptotic thymocytes could directly influence *Foxn1* expression in TECs. To do this we induced pyroptosis in freshly isolated thymocytes ex vivo using Nigericin (an NLRP3 activator [47]) and LPS (to provide a priming signal for NLRP3 activation [48]) and subsequently co-cultured the dying cells with cortical thymic epithelial cells (cTECs, using the C9 and ANV42.1 cell lines) and medullary thymic epithelial cells (mTEC, using the TE-71 cell line), and measured *Foxn1* expression (Fig. 3A). Using this approach, we demonstrated that the presence of pyroptotic thymocytes promotes increased *Foxn1* transcription in cTECs but not in mTECs (Fig. 3A), consistent with our previous observations that Foxn1 regulation during endogenous thymic regeneration is predominantly restricted to cTECs [11].

Pyroptotic cells release a plethora of molecules that act as ligands and messengers to facilitate communication with neighboring cells [38, 49, 50]. We have previously shown that extracellular Zn^2+^ can act as a damage-associated molecular pattern (DAMP) after acute damage, inducing expression of the pro-regenerative molecule BMP4 in endothelial cells via the receptor GPR39, although activation of GPR39 on TECs failed to induce *Foxn1* expression [51]. Therefore, we sought to refine our targets by identifying specific DAMPs that could trigger the induction of *Foxn1* transcription, specifically focusing on cTECs. To this end, we carried out an in vitro screen examining the response of the cTEC cell line (C9) to a panel of DAMPs and identified ATP to be a strong inducer of *Foxn1* transcription (Fig. 3B). This finding was confirmed in another cTEC cell line (ANV42.1) (Fig. 3C) and in freshly isolated TECs from human thymic tissue (Fig. 3D). We further examined the extracellular fluid from the damaged thymus and identified a significant increase in the amount of extracellular ATP in the thymus early after TBI (Fig. 3E). To examine the effect of ATP on FOXN1 protein levels in cTECs we stimulated primary thymus cells ex vivo with ATP and identified an increase in FOXN1+ cTECs, but not mTECs (Fig. 3F and Supplementary Fig. 1B), consistent with our hypothesis and highlighting cTECs as the major responders to ATP.

### ATP driven *Foxn1* transcription is mediated by activation of P2Y purinergic receptors

**Fig. 4:**
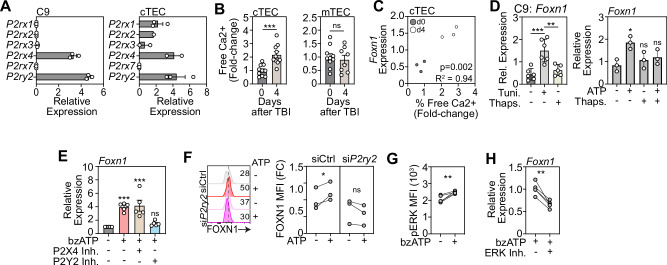
ATP-driven induction of Foxn1 is mediated by P2 purinergic receptors. **A** Purinergic receptor expression in FACS purified cTECs from untreated mice measured by qPCR (n = 2–3 pooled mouse thymuses); **B** Intracellular free Ca^2+^ levels were measured by flow cytometry in untreated and TBI-treated (day 4 post TBI) cTECs (n = 10 mice from 2 separate experiments) and mTECs (n = 8–10 mice from 2 separate experiments); **C** Correlation of free Ca^2+^ and *Foxn1* expression at days 0 and 4 after SL-TBI (n = 3/timepoint); **D** C9 (cTEC) were treated with tunicamycin (1 µM), thapsigargan (100 nM), or combined bzATP (100 µM) and thapsigargan (100 nM) for 20 h and *Foxn1* expression was measured by qPCR (n = 4 independent experiments); **E** C9 (cTECs) were treated with bzATP and either antagonists for P2Y2 or P2X4 and *Foxn1* expression was measured by qPCR 20 h after incubation (n = 5 separate experiments); **F** C9 cells were transfected with an siRNA target P2Y2 or a negative control siRNA and treated with bzATP (100 µM) or BMP4 (25 ng/ml) 3 days following transfection. FOXN1 levels were assessed by flow cytometry 18 h post ATP treatment (n = 3 independent experiments, one-way ANOVA, Dunn’s post hoc test); **G** C9 (cTECs) were treated with bzATP and ERK phosphorylation was assessed by flow cytometry (n = 5 individual experiments); **H** C9 (cTECs) were treated for 24 h with bzATP with or without an ERK inhibitor and *Foxn1* was measured by qPCR (n = 4 independent experiments). Summary data represent mean ± SEM. *, p < 0.05; **, p < 0.01; ***, p < 0.001.

ATP is a ligand for cell surface purinergic receptors and can activate downstream signaling pathways that either promote the influx of extracellular Ca^2+^ or the efflux of ER Ca^2+^ via G-coupled signaling [52–54]. Previous studies have shown that purinergic receptor expression is heterogeneous between thymic epithelial cell subsets, with widespread expression of both P2Y and P2X receptors expressed among all subsets of TECs [55]. Consistent with this, we confirmed baseline expression levels of multiple purinergic receptors in the C9 (cTEC cell line), and on freshly isolated murine cTECs (Fig. 4A).

Next, as P2 receptor activation signals downstream to increase intracellular Ca^2+^ levels, we measured Ca^2+^ levels in cTECs and mTECs after damage and identified increased Ca^2+^ levels in cTECs but not mTECs (Fig. 4B), which positively correlated with *Foxn1* expression (Fig. 4C). This data is consistent with the cTEC-specific effects of pyroptotic thymocytes on *Foxn1* expression, suggesting cTECs are central gatekeepers of the ATP-mediated regenerative response. Given that P2X and P2Y receptors regulate Ca^2+^ levels via distinct mechanisms [52, 56–58], we sought to examine the effect of ATP on both Ca^2+^ influx and efflux within cTECs. To do this we treated cTECs with tunicamycin, to induce ER release of Ca^2+^ into the cytosol, or thapsigargan, to inhibit ER Ca^2+^ release, and revealed that flooding the cell with Ca^2+^ led to enhanced *Foxn1* expression, while attenuating Ca^2+^ levels restored *Foxn1* expression to baseline (Fig. 4D). We additionally demonstrated that the combined treatment of ATP with thapsigargan attenuated Foxn1 expression (Fig. 4D), further strengthening the finding of a positive regulatory role for Ca²⁺ in promoting *Foxn1* expression.

To further refine our target and identify the specific purinergic receptor mediating this effect, we treated cTECs with ATP in the presence of antagonists targeting P2Y2 and P2X4. Although P2X4 is highly expressed on cTECs, it does not induce Ca^2+^ efflux. In contrast, specific inhibition of P2Y2 significantly attenuated ATP-induced *Foxn1* expression (Fig. 4E), supporting a key role for P2Y2 in mediating the ATP-driven regenerative signal. To strengthen these findings, we knocked down *P2ry2* in C9 cells using an siRNA and subsequently stimulated the cells with ATP. We observed that ATP-induced FOXN1 levels were attenuated when *P2ry2* was silenced (Fig. 4F and Supplementary Fig. 1C), further confirming the role of P2Y2 as a critical mediator of this regenerative signaling pathway.

Finally, as ERK phosphorylation is a key event in P2Y2 signal transduction [59], we examined ERK phosphorylation and found a significant increase in ERK phosphorylation following ATP stimulation, with the inhibition of ERK abrogated ATP-mediated increase in *Foxn1* (Fig. 4G, H), further substantiating the role of the P2Y2–Ca²⁺–ERK axis in regulating thymic epithelial cell regeneration.

### Activation of P2Y2 receptors enhances *Foxn1* expression and enhances thymus regeneration after acute injury

**Fig. 5:**
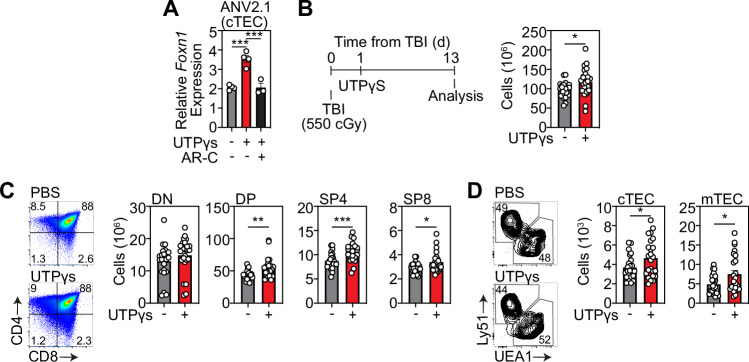
Activation of the P2Y2 enhances thymus regeneration after damage in vivo. **A** ANV42.1 (cTEC) cells were treated with the P2Y2 agonist UTPγS and the P2Y2 antagonist ARC118925XX for 20 h and *Foxn1* expression was measured by qPCR after 20 h (n = 3 separate experiments); **B**–**D** 6wo C57BL/6 mice were treated with UTPyS (1 mg/kg) IP at day 1 following SL-TBI and the thymuses were harvested at day 13. **B** Total thymus cellularity. **C** Number of CD4-CD8- double negative (DN), DP, of CD4 or CD8+ single positive (SP4 or SP8, respectively) thymocytes (n = 23–24 mice from 3 independent experiments). Summary data represent mean ± SEM. *, p < 0.05; **, p < 0.01; ***, p < 0.001.

P2 antagonists have gained momentum as attractive therapeutic targets, with a growing interest on developing agonists and antagonists to modulate their activity in a range of disease contexts, such as epilepsy [60], rheumatoid arthritis [61], and ischemic cardiac injury [62]. Several clinical trials have been carried out using antagonists for P2X3 [63], P2X7 [64, 65], and P2Y12 [66]. To evaluate if P2Y2 could be targeted to enhance TEC functio,n we obtained a specific P2Y2 agonist and further demonstrated that stimulation of cTECs with a P2Y2 agonist induced *Foxn1* expression, while pharmacological inhibition of P2Y2 ablated this response (Fig. 5A), confirming receptor specificity. To test the therapeutic potential of targeting P2Y2 on enhancing thymic function, we carried out in vivo experiments administering the P2Y2 agonist UTPγS to C57BL/6 mice on day 1 following damage (SL-TBI). We demonstrated that ice treated with UTPγS exhibited enhanced thymic cellularity 13 days post damage, compared with control treated mice (Fig. 5B), indicating that P2Y2 activation promotes thymic regeneration following acute damage. Furthermore, UTPγS treatment had a broad impact on thymocyte reconstitution, with increased regeneration of DPs, CD4+, and CD8+ thymocytes. Importantly, there was a marked enhancement in the regeneration of TECs following UTPγS treatment (Fig. 5C), consistent with our in vitro studies and further supporting the regenerative potential of P2Y2 agonism after acute damage.

## Discussion

Endogenous thymic regeneration is crucial for effective T cell reconstitution after HCT, but it relies on complex multicellular signaling within specialized thymic cellular niches. In this study, we examined how HCT conditioning reshapes thymocyte cell fate and metabolic landscape. We identified a cell-specific mechanism of thymus regeneration centered the acute induction of lytic cell death in DP thymocytes, facilitated by transient and aberrant mitochondrial responses which position thymocytes to undergo downstream caspase-1 cleavage and pyroptotic cell death.

In contrast to canonical apoptosis, which is immunologically silent [67], lytic cell death, such as pyroptosis, exposes a vast range of ligands that can influence immune function [68]. Notably, strategies targeting pyroptotic cell death have emerged as therapeutically promising in a range of disease settings, such as several cancers [69, 70] and type II diabetes [71], in addition to their role in sterile inflammatory conditions such as asthma [72]. We have previously shown that zinc, which increases in thymocytes during their development, accumulates in the thymic microenvironment after acute damage and promotes regeneration by initiating an increase in levels of pro-regenerative factors such as BMP4 [51]. Here, we have identified another key damage-induced molecule, ATP, which mediates its pro-regenerative effects directly on TECs through the activation of the cell-surface purinergic receptor P2Y2, leading to upregulation of the key thymic transcription factor FOXN1.

Both intracellular and extracellular ATP have been implicated in tissue repair [73–75], and pharmacologic targeting of ATP-activated purinergic receptors are being explored in wound repair [75–77]. Although antagonism of P2X7 showed promise in preclinical models, Phase II clinical trials in rheumatoid arthritis failed to translate efficacy [65, 78]. However, considerable interest remains in therapeutically targeting purinergic receptors, with P2X7 antagonists being tested to treat Crohn's disease, and the commercial P2Y2 agonist Diquafosol is currently being tested for the treatment of dry eye [79]. Previous studies identified that activation of purinergic receptors mobilizes intracellular Ca^2+^ in epithelial cells and has been found to trigger their repair [80, 81], which is consistent with our data demonstrating that inhibiting Ca^2+^ efflux from the ER using thapsigargin prevented FOXN1 transcription. Further, we identified that P2Y2 agonism promotes FOXN1 transcription specifically in cTECs and that competition with an antagonist quenches this effect, pointing to receptor specificity, although further studies are required to identify if other ATP-sensing purinergic receptors can also mediate this effect in vivo.

Mitochondrial destabilization has been linked to lytic cell death [82]. Our data indicate that DP thymocytes are metabolically sensitized for pyroptotic cell death following acute injury, rapidly activating caspase-1 to initiate a regeneration cascade. The thymus is hypoxic at steady-state [83], and thymocytes undergo dynamic alterations in metabolic activity during development, specifically between DN and DP stages, pointing to their metabolic plasticity [33, 84]. Multiple studies have identified ROS to interact with the NLRP3 inflammasome and drive pyroptotic cell death [15, 85–88]. Here, we identified that mitochondrial dysfunction precedes pyroptotic-mediated regeneration of the thymus and demonstrate that altered cellular pyruvate:lactate, together with increased mitochondrial ROS, primes DP thymocytes for caspase-1 cleavage and downstream pyroptosis. This raises the important question of how nutrient sources - such as glucose, lipids, or glutamine - and metabolic state shape mitochondrial dysfunction during acute damage and may uncover disease-specific damage signatures of the regenerative response. Notably, lipid metabolism has been implicated in both hematopoiesis and T cell differentiation [89], suggesting it may play a central role in modulating thymic regeneration following injury.

Together, these data describe a complex molecular network that governs thymus regeneration and provides a platform for therapeutic target discovery aimed at enhancing immune reconstitution. Moreover, these findings contribute to the broader field of regenerative medicine by uncovering novel mechanisms of damage-induced endogenous tissue repair that may extend beyond the thymus to other organ systems.

## Materials and methods

### Mice

Inbred male and female C57BL/6J mice were obtained from the Jackson Laboratories (Bar Harbor, USA) and all experimental mice were used between 6 and 8 weeks old. To induce thymic damage, mice were given sub-lethal total body irradiation (SL-TBI) at a dose of 550 cGy from a cesium source mouse irradiator (Mark I series 30JL Shepherd irradiator) with no hematopoietic rescue. Mice were maintained at the Fred Hutchinson Cancer Research Center (Seattle, WA) and acclimatized for at least 2 days before experimentation, which was performed per Institutional Animal Care and Use Committee guidelines.

### Antibodies and reagents

Cells were stained with the following antibodies for analysis CD3-FITC (35-0031, Tonbo Bioscience), CD8-BV711 (100748, BioLegend), CD4-BV650 (100546, BioLegend), CD45BUV395 (565967, BD Biosciences), CD90-BV785 (105331, BioLegend), MHC-II-Pac Blue (107620, BioLegend), EpCAM-PercPe710 (46-5791-82, eBioscience), Ly51-PE (12-5891-83, eBioscience), UEA1-FITC (FL-1061, Vector Laboratories), TCRbeta-PECy7 (109222, BioLegend), CD62L-APC-Cy7 (104427, BioLegend), CD44-Alexa Fluor RTM700 (56-0441-82, BioLegend), CD25-PercP-Cy5.5 (102030, BioLegend), TER119 BV711 (Biolegend, 116267), CD45 BUV395 (BD Bioscience, 565967), FOXN1 antibody (Invitrogen, PA5-21618), secondary APC-conjugated antibody (Cell Signaling, 4414S), and mouse Mouse FcR Blocker (Stemcell, 18730). Foxp3/Transcription Factor Staining Buffer Set (Invitrogen, 00-5523-00) was used for FOXN1 staining. Flow cytometry analysis was performed on an LSRFortessa X50 (BD Biosciences) and cells were sorted on an Aria II (BD Biosciences) using FACSDiva (BD Biosciences) or FlowJo (Treestar Software).

### Thymus digestion and cell isolation

Single cell suspensions of freshly dissected thymuses were obtained and either mechanically suspended or enzymatically digested as previously described [8, 27] and counted using the Z2 Coulter Particle and Size Analyzer (Beckman Coulter, USA). For studies sorting rare populations of cells in the thymus, multiple identically treated thymuses were pooled so that a sufficient number of cells could be isolated; however, in this instance separate pools of cells were established to maintain individual samples as biological replicates.

### Cell death assays

Thymuses from untreated and SL-TBI-treated mice were harvested, enzymatically digested, and stained with cell surface markers for thymus populations. Cells were further stained for caspase 1 cleavage with Caspase-1 (active) Staining Kit (Abcam, ab219935), or fixed for caspase 3 analysis using Cleaved Caspase-3 (Asp175) Antibody (Alexa Fluor® 488 Conjugate) (Cell Signaling, 9669S). Apoptosis and pyroptosis were assessed by adding Annexin V-FITC (Biolegend, 640906), Annexin V binding buffer (BioLegend, 422201,) and Propidium Iodide (Invitrogen, BMS500PI). Gasdermin D was measured in freshly isolated thymocytes using Gasdermin D (mouse) ELISA Kit (Adipogen Life Sciences, AG-45B-0011-KI01). Lactate dehydrogenase was assessed from the supernatant of harvested thymocytes using the Lactate Dehydrogenase assay (Abcam, ab102526).

### Western blotting

Thymus cell lysates were obtained by homogenizing the thymii at 50 mg/ml in RIPA buffer with protease inhibitors (Pierce #A32955). The samples were reduced and run on a BioRad 4–20% mini-Protean TGX gel and the gel was transferred to PVDF membrane and blocked with 2.0% NFDM, 150 mM NaCl, 50 mM Tris pH 7.4, and 0.05% Tween for 2 h. The blot was probed with anti-cleaved Gasdermin D antibody (CST #50928S) or anti-cleaved caspase 1 p20 antibody (AdipoGen Life Sciences #AG-20B-0042 cat number) at 1:1000 overnight in blocking buffer with 1% NFDM and subsequently incubated with Li-COR IRDye 800CW Goat anti-Rabbit at 1:20,000. Bands were detected using the Li-COR Odyssey CLx.

### In vitro assays

#### Co-culture assays

Thymocytes were isolated from untreated C57BL/6 mice and incubated with Nigericin (10 µM, Tocris, 4312) and LPS (1 ng/ml, Invivogen, tlrl-eblps) for 3 h and co-cultured with C9s, ANV42.1 or TE-71 cell lines for 20 h before lysis for qPCR. *DAMP stimulation:* C9s were stimulated with ATP (100 µM, Tocris 3312), HMGB1 (1 µg/ml, Abcam, ab78940), IL1a (50 ng/ml, Tocris, 400-ML-005/CF), IL-33 (50 ng/ml, Tocris, 3626-ML-010/CF), uric acid (50 µg/ml, Sigma, U2625), dsDNA (370 ng), or mtDNA (370 ng) for 20 h and lysed for qPCR analysis. *UTPγS assays:* C9s or ANV42, cells were stimulated with UTPγS (100 µM, R&D Systems, 3279), or UTPγS plus AR-C 118925XX (20 µM, Tocris, 4890) for 20 h before lysis for qPCR. BzATP triethylammonium salt (100-300 µM, Tocris, 3312) was used for ATP stimulation. dsDNA was isolated from C9s using a DNeasy Blood and Tissue kit (Qiagen 69504), and mtDNA was isolated from C9s using a Mitochondrial DNA Isolation Kit (Abcam ab65321).

#### Thapsigargan treatment

C9s were plated at 5 × 10^5^ cells/ well in a 24-well plate and treated with 100 µM bzATP or 100 nM thapsigargin for 20 h and lysed for RNA extraction. *P2Y2 silencing assay:* C9 cells were plated at 5,000 cells/well on a 96-well plate and transfected with 50 nM Silencer Negative Control siRNA (Invitrogen, AM4641) or P2Y2 siRNA (Invitrogen, 4390815) using Lipofectamine RNAiMAX (Invitrogen, 2026-12-06). The cells were treated with 100 µM BzATP or 25 ng/ml BMP4 for 18 h at 3 days post transfection and subsequently stained using the following panel: anti-UEA1 FITC, anti-EpCAM PE, anti-PDGR1aPE Texas Red, anti-CD45 BUV395, anti-MHC-II BV421, anti-Ly51 BV786, anti-CD31 PE-Cy7, anti-TER119 BV711, The cells were fixed using the Foxp3/Transcription Factor Staining Buffer Set (Invitrogen, 00-5523-00) and stained with anti-FOXN1 (Invitrogen, PA5-21618) at 1:200 for 30 min at room temperature. The cells were further stained with anti-IgG Alexa 647 (Cell Signaling, 4414S) at 1:500 for 30 min at room temperature. The cells were washed twice with PBS and analyzed by flow cytometry.

### qPCR

RNA was extracted from cells using a RNeasy Mini kit (74104, Qiagen), and from sorted cells using a RNeasy Plus Micro kit (74034, Qiagen). cDNA was synthesized using the iScript gDNA Clear cDNA Synthesis kit (1725035, Bio-Rad, USA) and a Bio-Rad C1000 Touch ThermoCycler (Bio-Rad). RNA expression was assessed in the Bio-Rad CFX96 Real Time System (Bio-Rad), using iTaq Universal SYBR Green Supermix (1725122, Bio-Rad), and the following primers: β-Actin (F 5’-CACTGTCGAGTCGCGTCC-3’; R 5’-TCATCCATGGCGAACTGGTG-3’); PrimePCR™ SYBR® Green Assay: Foxn1, Mouse (Biorad, 10025637, qMmuCED0044924); P2Y2 (Invitrogen, F 5’-GGCAACAGCACGTACTTGAA-3’; R 5’CAGGCCTGTGCATATGTGAG-3’); P2X1 (Invitrogen, F 5’ CCTGGGAGGCTGATCAACAG-3’; R 5’-GTGTGGCAGGAGAACAC-3’); P2X4 (Invitrogen, F 5’-TATGTGGTCCCAGCTCAGGA-3’; R 5’-TCACAGACGCGTTGATTGGA-3’); P2X7 (Invitrogen, F 5’-GGGGGTTTACCCCTACTGTA-3’; R 5’-GCTCGTCGACAAAGGACAC-3’).

### Ex vivo assays

#### Metabolic assays

Thymuses from untreated and SL-TBI-treated mice were harvested and enzymatically digested and stained from flow cytometry analysis of thymocyte populations as above. Further analysis of mitochondrial bioenergetics was assessed using TMRE (Abcam, ab113852), MitoTracker™ Green FM (Invitrogen, M7514), MitoSOX™ Red Mitochondrial Superoxide Indicator (ThermoFisher, M36008), and Intracellular Glutathione (GSH) Detection Assay Kit (Abcam, ab112132). Thymocytes were isolated from untreated and TBI-treated mice and intracellular pyruvate and lactate levels were measured by absorbance using Pyruvate Assay kit (Abcam, ab65342) or Lactate-Glo™ Assay (Promega, J5021). Thymocytes were isolated from untreated and TBI-treated mice and incubated in RPMI with 5 mM sodium pyruvate (Gibco, 11360070) plus 200 µM α-ketobutyrate (Sigma-Aldrich) for 3 h at 37 °C. *ATP stimulation* ex vivo: Thymuses were harvested from 8-week-old female untreated C57BL/6J mice and enzymatically digested as above. The cells were plated at 2 × 10^6^ cells/well in a 24-well plate and treated with 100 µM bzATP or 25 ng/ml BMP4 for 18 h and prepared for flow cytometry.

### Intracellular Ca^2+^ assay

Thymuses were harvested from untreated and SL-TBI-treated mice and processed for flow cytometry as described above. The intracellular Ca^2+^ dye BAPTA-AM/Indo-AM was added (Sigma-Aldrich). Unbound intracellular Ca^2+^ was assessed in cTECs and mTECs by measuring BAPTA-AM levels on the BUV-496 filter.

### In vivo UTPγS administration

For in vivo studies of UTPγS administration, mice were given SL-TBI (550 cGy) and subsequently received intraperitoneal injections of 1 mg/kg UTPγS (R&D systems, 3279), or 1× PBS as control, on day 1 following TBI. Thymuses were harvested 13 days after SL-TBI and cellularity was assessed and populations were analyzed by flow cytometry.

### Statistical analysis

All analysis between two groups was performed with a non-parametric Mann-Whitney test. Statistical comparison between 3 or more groups in Figs. 1A, C, D, E, F, G, 2A, B, C, E, 3A, H, I, and 4A were performed using a one-way ANOVA with Tukey’s multiple comparison post-hoc test. All statistics were calculated using Graphpad Prism and display graphs were generated in Graphpad Prism.

## Summary

- Thymocytes rapidly undergo pyroptotic cell death after acute thymic damage and release ATP.
- Extracellular ATP release from thymocytes promotes *Foxn1* expression in cTECs via activation of P2Y2 and drives thymus regeneration.

## Supplementary information

Supplementary Figure 1

Uncropped Western Blots

## Data Availability

Any data and materials that can be shared will be released via a Material Transfer Agreement.

## References

[1] Kinsella S, Dudakov JA. When the damage is done: injury and repair in thymus function. Front Immunol. 2020;11:1745. 32903477 10.3389/fimmu.2020.01745 PMC7435010

[2] Admiraal R, de Koning CCH, Lindemans CA, Bierings MB, Wensing AMJ, Versluys AB, et al. Viral reactivations and associated outcomes in the context of immune reconstitution after pediatric hematopoietic cell transplantation. J Allergy Clin Immunol. 2017;140:1643–50.e9. 28392330 10.1016/j.jaci.2016.12.992

[3] de Koning C, Prockop S, van Roessel I, Kernan N, Klein E, Langenhorst J, et al. CD4+ T-cell reconstitution predicts survival outcomes after acute graft-versus-host-disease: a dual-center validation. Blood. 2021;137:848–55. 33150379 10.1182/blood.2020007905 PMC7986048

[4] Chinn IK, Blackburn CC, Manley NR, Sempowski GD. Changes in primary lymphoid organs with aging. Semin Immunol. 2012;24:309–20. 22559987 10.1016/j.smim.2012.04.005 PMC3415579

[5] Granadier D, Iovino L, Kinsella S, Dudakov JA. Dynamics of thymus function and T cell receptor repertoire breadth in health and disease. Semin Immunopathol. 2021;43:119–34. 33608819 10.1007/s00281-021-00840-5 PMC7894242

[6] Kousa AI, Jahn L, Zhao K, Flores AE, Acenas D 2nd, Lederer E, et al. Age-related epithelial defects limit thymic function and regeneration. Nat Immunol. 2024;25:1593–606. 39112630 10.1038/s41590-024-01915-9 PMC11362016

[7] van den Broek T, Delemarre EM, Janssen WJM, Nievelstein RAJ, Broen JC, Tesselaar K, et al. Neonatal thymectomy reveals differentiation and plasticity within human naive T cells. J Clin Investig. 2016;126:1126–36. 26901814 10.1172/JCI84997 PMC4767338

[8] Dudakov JA, Hanash AM, Jenq RR, Young LF, Ghosh A, Singer NV, et al. Interleukin-22 drives endogenous thymic regeneration in mice. Science. 2012;336:91–5. 22383805 10.1126/science.1218004 PMC3616391

[9] Goldberg GL, Dudakov JA, Reiseger JJ, Seach N, Ueno T, Vlahos K, et al. Sex steroid ablation enhances immune reconstitution following cytotoxic antineoplastic therapy in young mice. J Immunol. 2010;184:6014–24. 20483779 10.4049/jimmunol.0802445

[10] Gui J, Mustachio LM, Su DM, Craig RW. Thymus size and age-related thymic involution: early programming, sexual dimorphism, progenitors and stroma. Aging Dis. 2012;3:280–90. 22724086 PMC3375084

[11] Wertheimer T, Velardi E, Tsai J, Cooper K, Xiao S, Kloss CC, et al. Production of BMP4 by endothelial cells is crucial for endogenous thymic regeneration. Sci Immunol. 2018;3:eaal2736. 10.1126/sciimmunol.aal2736 PMC5795617 29330161

[12] Vaidya HJ, Briones Leon A, Blackburn CC. FOXN1 in thymus organogenesis and development. Eur J Immunol. 2016;46:1826–37. 27378598 10.1002/eji.201545814 PMC4988515

[13] Kinsella S, Evandy CA, Cooper K, Iovino L, deRoos PC, Hopwo KS, et al. Attenuation of apoptotic cell detection triggers thymic regeneration after damage. Cell Rep. 2021;37:109789. 34610317 10.1016/j.celrep.2021.109789 PMC8627669

[14] Jeong SY, Seol DW. The role of mitochondria in apoptosis. BMB Rep. 2008;41:11–22. 18304445 10.5483/bmbrep.2008.41.1.011

[15] Wang Y, Shi P, Chen Q, Huang Z, Zou D, Zhang J, et al. Mitochondrial ROS promote macrophage pyroptosis by inducing GSDMD oxidation. J Mol Cell Biol. 2019;11:1069–82. 30860577 10.1093/jmcb/mjz020 PMC6934151

[16] Glover HL, Schreiner A, Dewson G, Tait SWG. Mitochondria and cell death. Nat Cell Biol. 2024;26:1434–46. 38902422 10.1038/s41556-024-01429-4

[17] Vringer E, Tait SWG. Mitochondria and cell death-associated inflammation. Cell Death Differ. 2023;30:304–12. 36447047 10.1038/s41418-022-01094-w PMC9950460

[18] Venereau E, Ceriotti C, Bianchi ME. DAMPs from Cell Death to New Life. Front Immunol. 2015;6:422–422. 26347745 10.3389/fimmu.2015.00422 PMC4539554

[19] Anders H-J, Schaefer L. Beyond tissue injury-damage-associated molecular patterns, toll-like receptors, and inflammasomes also drive regeneration and fibrosis. J Am Soc Nephrol. 2014;25:1387–400. 24762401 10.1681/ASN.2014010117 PMC4073442

[20] Yang R, Tonnesseen TI. DAMPs and sterile inflammation in drug hepatotoxicity. Hepatol Int. 2019;13:42–50. 30474802 10.1007/s12072-018-9911-9

[21] Wilgus TA. Alerting the body to tissue injury: the role of alarmins and DAMPs in cutaneous wound healing. Curr Pathobiol Rep. 2018;6:55–60. 29862143 10.1007/s40139-018-0162-1 PMC5978745

[22] Simader E, Beer L, Laggner M, Vorstandlechner V, Gugerell A, Erb M, et al. Tissue-regenerative potential of the secretome of γ-irradiated peripheral blood mononuclear cells is mediated via TNFRSF1B-induced necroptosis. Cell Death Dis. 2019;10:729. 31570701 10.1038/s41419-019-1974-6 PMC6768878

[23] Klemm J, Stinchfield MJ, Harris RE. Necrosis-induced apoptosis promotes regeneration in Drosophila wing imaginal discs. Genetics. 2021;219:iyab144. 10.1093/genetics/iyab144 PMC8570793 34740246

[24] Granadier D, Cooper K, Acenas D, Kousa A, Warren M, Hernandez V, et al. Damage-induced IL-18 stimulates thymic NK cells limiting endogenous tissue regeneration. Nat Immunol. 2025;26:1699–1711. 40935830 10.1038/s41590-025-02270-z PMC12479347

[25] Tackett BC, Sun H, Mei Y, Maynard JP, Cheruvu S, Mani A, et al. P2Y2 purinergic receptor activation is essential for efficient hepatocyte proliferation in response to partial hepatectomy. Am J Physiol Gastrointest Liver Physiol. 2014;307:G1073–87. 25301185 10.1152/ajpgi.00092.2014 PMC4254960

[26] Cheng R, Zhu G, Ni C, Wang R, Sun P, Tian L, et al. P2Y2 Receptor mediated neuronal regeneration and angiogenesis to affect functional recovery in rats with spinal cord injury. Neural Plast. 2022;2022:2191011. 35154311 10.1155/2022/2191011 PMC8828345

[27] Yang Y, Ashwell JD. Thymocyte apoptosis. J Clin Immunol. 1999;19:337–49. 10634208 10.1023/a:1020594531159

[28] Erlacher M, Labi V, Manzl C, Böck GN, Tzankov A, Häcker G, et al. Puma cooperates with Bim, the rate-limiting BH3-only protein in cell death during lymphocyte development, in apoptosis induction. J Exp Med. 2006;203:2939–51. 17178918 10.1084/jem.20061552 PMC2118188

[29] Cairns JS, Mainwaring MS, Cacchione RN, Walker JA, McCarthy SA. Regulation of apoptosis in thymocytes. Thymus. 1993;21:177–93. 8236376

[30] Erlacher M, Michalak EM, Kelly PN, Labi V, Niederegger H, Coultas L, et al. BH3-only proteins Puma and Bim are rate-limiting for γ-radiation– and glucocorticoid-induced apoptosis of lymphoid cells in vivo. Blood. 2005;106:4131–8. 16118324 10.1182/blood-2005-04-1595 PMC1895232

[31] Vringer E, Tait SWG. Mitochondria and inflammation: cell death heats up. Front Cell Dev Biol. 2019;7:100. 31316979 10.3389/fcell.2019.00100 PMC6610339

[32] Li Q, Shi N, Cai C, Zhang M, He J, Tan Y, et al. The role of mitochondria in pyroptosis. Front Cell Dev Biol. 2020;8:630771. 33553170 10.3389/fcell.2020.630771 PMC7859326

[33] Sun V, Sharpley M, Kaczor-Urbanowicz KE, Chang P, Montel-Hagen A, Lopez S, et al. The metabolic landscape of thymic T cell development in vivo and in vitro. Front Immunol. 2021;12:716661. 34394122 10.3389/fimmu.2021.716661 PMC8355594

[34] Shi Y, Zhang H, Miao C. Metabolic reprogram and T cell differentiation in inflammation: current evidence and future perspectives. Cell Death Discov. 2025;11:123. 40155378 10.1038/s41420-025-02403-1 PMC11953409

[35] Zhang M, Lin X, Yang Z, Li X, Zhou Z, Love PE, et al. Metabolic regulation of T cell development. Front Immunol. 2022;13:946119. 35958585 10.3389/fimmu.2022.946119 PMC9357944

[36] Camilli G, Bohm M, Piffer AC, Lavenir R, Williams DL, Neven B, et al. β-Glucan-induced reprogramming of human macrophages inhibits NLRP3 inflammasome activation in cryopyrinopathies. J Clin Invest. 2020;130:4561–73. 32716363 10.1172/JCI134778 PMC7456248

[37] Wu X, Zhang H, Qi W, Zhang Y, Li J, Li Z, et al. Nicotine promotes atherosclerosis via ROS-NLRP3-mediated endothelial cell pyroptosis. Cell Death Dis. 2018;9:171. 29416034 10.1038/s41419-017-0257-3 PMC5833729

[38] Man SM, Karki R, Kanneganti TD. Molecular mechanisms and functions of pyroptosis, inflammatory caspases and inflammasomes in infectious diseases. Immunol Rev. 2017;277:61–75. 28462526 10.1111/imr.12534 PMC5416822

[39] Zhao S, Chen F, Yin Q, Wang D, Han W, Zhang Y. Reactive oxygen species interact With NLRP3 inflammasomes and are involved in the inflammation of sepsis: from mechanism to treatment of progression. Front Physiol. 2020;11:571810. 33324236 10.3389/fphys.2020.571810 PMC7723971

[40] Pereira CA, Carlos D, Ferreira NS, Silva JF, Zanotto CZ, Zamboni DS, et al. Mitochondrial DNA promotes NLRP3 inflammasome activation and contributes to endothelial dysfunction and inflammation in type 1 diabetes. Front Physiol. 2019;10:1557. 32009974 10.3389/fphys.2019.01557 PMC6978691

[41] Liu Q, Zhang D, Hu D, Zhou X, Zhou Y. The role of mitochondria in NLRP3 inflammasome activation. Mol Immunol. 2018;103:115–24. 30248487 10.1016/j.molimm.2018.09.010

[42] Pi S, Nie G, Wei Z, Yang F, Wang C, Xing C, et al. Inhibition of ROS/NLRP3/Caspase-1 mediated pyroptosis alleviates excess molybdenum-induced apoptosis in duck renal tubular epithelial cells. Ecotoxicol Environ Saf. 2021;208:111528. 33157513 10.1016/j.ecoenv.2020.111528

[43] Kwon DH, Cha HJ, Lee H, Hong SH, Park C, Park SH, et al. Protective effect of glutathione against oxidative stress-induced cytotoxicity in RAW 264.7 macrophages through activating the nuclear factor erythroid 2-related factor-2/heme oxygenase-1 pathway. Antioxidants=. 2019;8:82. 10.3390/antiox8040082 PMC6523540 30939721

[44] Liu T, Sun L, Zhang Y, Wang Y, Zheng J. Imbalanced GSH/ROS and sequential cell death. J Biochem Mol Toxicol. 2022;36:e22942. 34725879 10.1002/jbt.22942

[45] Lapointe DS, Olson MS. alpha-Ketobutyrate metabolism in perfused rat liver: regulation of alpha-ketobutyrate decarboxylation and effects of alpha-ketobutyrate on pyruvate dehydrogenase. Arch Biochem Biophys. 1985;242:417–29. 4062289 10.1016/0003-9861(85)90226-7

[46] Brass EP. Effect of alpha-ketobutyrate on palmitic acid and pyruvate metabolism in isolated rat hepatocytes. Biochim Biophys Acta. 1986;888:18–24. 3741887 10.1016/0167-4889(86)90065-0

[47] Armstrong H, Bording-Jorgensen M, Chan R, Wine E. Nigericin promotes NLRP3-independent bacterial killing in macrophages. Front Immunol. 2019;10:2296. 31632394 10.3389/fimmu.2019.02296 PMC6779719

[48] Bauernfeind FG, Horvath G, Stutz A, Alnemri ES, MacDonald K, Speert D, et al. Cutting edge: NF-kappaB activating pattern recognition and cytokine receptors license NLRP3 inflammasome activation by regulating NLRP3 expression. J Immunol. 2009;183:787–91. 19570822 10.4049/jimmunol.0901363 PMC2824855

[49] Chen CJ, Kono H, Golenbock D, Reed G, Akira S, Rock KL. Identification of a key pathway required for the sterile inflammatory response triggered by dying cells. Nat Med. 2007;13:851–6. 17572686 10.1038/nm1603

[50] Broz P. Pyroptosis: molecular mechanisms and roles in disease. Cell Res. 2025;35:334–44. 40181184 10.1038/s41422-025-01107-6 PMC12012027

[51] Iovino L, Cooper K, deRoos P, Kinsella S, Evandy C, Ugrai T, et al. Activation of the zinc-sensing receptor GPR39 promotes T-cell reconstitution after hematopoietic cell transplant in mice. Blood. 2022;139:3655–66. 35357432 10.1182/blood.2021013950 PMC9227099

[52] Dubyak GR, el-Moatassim C. Signal transduction via P2-purinergic receptors for extracellular ATP and other nucleotides. Am J Physiol. 1993;265:C577–606. 8214015 10.1152/ajpcell.1993.265.3.C577

[53] Abbracchio MP, Burnstock G, Boeynaems JM, Barnard EA, Boyer JL, Kennedy C, et al. International Union of Pharmacology LVIII: update on the P2Y G protein-coupled nucleotide receptors: from molecular mechanisms and pathophysiology to therapy. Pharmacol Rev. 2006;58:281–341. 16968944 10.1124/pr.58.3.3 PMC3471216

[54] May C, Weigl L, Karel A, Hohenegger M. Extracellular ATP activates ERK1/ERK2 via a metabotropic P2Y1 receptor in a Ca2+ independent manner in differentiated human skeletal muscle cells. Biochem Pharmacol. 2006;71:1497–509. 16533496 10.1016/j.bcp.2006.02.003

[55] Bisaggio RD, Nihei OK, Persechini PM, Savino W, Alves LA. Characterization of P2 receptors in thymic epithelial cells. Cell Mol Biol. 2001;47:19–31. 11292255

[56] Wang J, Yu Y. Insights into the channel gating of P2X receptors from structures, dynamics and small molecules. Acta Pharmacol Sin. 2016;37:44–55. 26725734 10.1038/aps.2015.127 PMC4722974

[57] Lovászi M, Branco Haas C, Antonioli L, Pacher P, Haskó G. The role of P2Y receptors in regulating immunity and metabolism. Biochem Pharmacol. 2021;187:114419. 33460626 10.1016/j.bcp.2021.114419

[58] Rueschpler L, Schloer S. Beyond the surface: P2Y receptor downstream pathways, TLR crosstalk and therapeutic implications for infection and autoimmunity. Pharmacol Res. 2025;219:107884. 40744153 10.1016/j.phrs.2025.107884

[59] Chang SJ, Tzeng CR, Lee YH, Tai CJ. Extracellular ATP activates the PLC/PKC/ERK signaling pathway through the P2Y2 purinergic receptor leading to the induction of early growth response 1 expression and the inhibition of viability in human endometrial stromal cells. Cell Signal. 2008;20:1248–55. 18434089 10.1016/j.cellsig.2008.02.011

[60] Alves M, Beamer E, Engel T. The metabotropic purinergic P2Y receptor family as novel drug target in epilepsy. Front Pharmacol. 2018;9:193. 29563872 10.3389/fphar.2018.00193 PMC5851315

[61] Gao F, Li X. P2Y11 receptor antagonist NF340 ameliorates inflammation in human fibroblast-like synoviocytes: an implication in rheumatoid arthritis. IUBMB Life. 2019;71:1552–60. 31301116 10.1002/iub.2077

[62] Feliu C, Peyret H, Poitevin G, Cazaubon Y, Oszust F, Nguyen P, et al. Complementary role of P2 and adenosine receptors in ATP induced-anti-apoptotic effects against hypoxic injury of HUVECs. Int J Mol Sci. 2019;20:1446. 10.3390/ijms20061446 PMC6470483 30909368

[63] Abdulqawi R, Dockry R, Holt K, Layton G, McCarthy BG, Ford AP, et al. P2X3 receptor antagonist (AF-219) in refractory chronic cough: a randomised, double-blind, placebo-controlled phase 2 study. Lancet. 2015;385:1198–205. 25467586 10.1016/S0140-6736(14)61255-1

[64] Rech JC, Bhattacharya A, Letavic MA, Savall BM. The evolution of P2X7 antagonists with a focus on CNS indications. Bioorg Med Chem Lett. 2016;26:3838–45. 27426304 10.1016/j.bmcl.2016.06.048

[65] Keystone EC, Wang MM, Layton M, Hollis S, McInnes IB. Clinical evaluation of the efficacy of the P2X7 purinergic receptor antagonist AZD9056 on the signs and symptoms of rheumatoid arthritis in patients with active disease despite treatment with methotrexate or sulphasalazine. Ann Rheum Dis. 2012;71:1630–5. 22966146 10.1136/annrheumdis-2011-143578

[66] Sarafoff N, Byrne RA, Sibbing D. Clinical use of clopidogrel. Curr Pharm Des. 2012;18:5224–39. 22724411 10.2174/138161212803251853

[67] Bertheloot D, Latz E, Franklin BS. Necroptosis, pyroptosis and apoptosis: an intricate game of cell death. Cell Mol Immunol. 2021;18:1106–21. 33785842 10.1038/s41423-020-00630-3 PMC8008022

[68] Yu P, Zhang X, Liu N, Tang L, Peng C, Chen X. Pyroptosis: mechanisms and diseases. Signal Transduct Target Ther. 2021;6:128. 33776057 10.1038/s41392-021-00507-5 PMC8005494

[69] Li L, Li Y, Bai Y. Role of GSDMB in pyroptosis and cancer. Cancer Manag Res. 2020;12:3033–43. 32431546 10.2147/CMAR.S246948 PMC7201009

[70] Huang CF, Chen L, Li YC, Wu L, Yu GT, Zhang WF, et al. NLRP3 inflammasome activation promotes inflammation-induced carcinogenesis in head and neck squamous cell carcinoma. J Exp Clin Cancer Res. 2017;36:116. 28865486 10.1186/s13046-017-0589-y PMC5581464

[71] Chang H, Chang H, Cheng T, Lee GD, Chen X, Qi K. Micro-ribonucleic acid-23a-3p prevents the onset of type 2 diabetes mellitus by suppressing the activation of nucleotide-binding oligomerization-like receptor family pyrin domain containing 3 inflammatory bodies-caused pyroptosis through negatively regulating NIMA-related kinase 7. J Diabetes Investig. 2021;12:334–45. 32881354 10.1111/jdi.13396 PMC7926233

[72] Zhuang J, Cui H, Zhuang L, Zhai Z, Yang F, Luo G, et al. Bronchial epithelial pyroptosis promotes airway inflammation in a murine model of toluene diisocyanate-induced asthma. Biomed Pharmacother. 2020;125:109925. 32014690 10.1016/j.biopha.2020.109925

[73] Sarojini H, Billeter AT, Eichenberger S, Druen D, Barnett R, Gardner SA, et al. Rapid tissue regeneration induced by intracellular ATP delivery-A preliminary mechanistic study. PLoS ONE. 2017;12:e0174899. 28380006 10.1371/journal.pone.0174899 PMC5381896

[74] Hill LM, Gavala ML, Lenertz LY, Bertics PJ. Extracellular ATP may contribute to tissue repair by rapidly stimulating purinergic receptor X7-dependent vascular endothelial growth factor release from primary human monocytes. J Immunol. 2010;185:3028–34. 20668222 10.4049/jimmunol.1001298 PMC3156583

[75] McEwan TB, Sophocleous RA, Cuthbertson P, Mansfield KJ, Sanderson-Smith ML, Sluyter R. Autocrine regulation of wound healing by ATP release and P2Y(2) receptor activation. Life Sci. 2021;283:119850. 34314735 10.1016/j.lfs.2021.119850

[76] Gendaszewska-Darmach E, Kucharska M. Nucleotide receptors as targets in the pharmacological enhancement of dermal wound healing. Purinergic Signal. 2011;7:193. 21519856 10.1007/s11302-011-9233-z PMC3146642

[77] Greig AV, James SE, McGrouther DA, Terenghi G, Burnstock G. Purinergic receptor expression in the regeneration epidermis in a rat model of normal and delayed wound healing. Exp Dermatol. 2003;12:860–71. 14714568 10.1111/j.0906-6705.2003.00110.x

[78] Stock TC, Bloom BJ, Wei N, Ishaq S, Park W, Wang X, et al. Efficacy and safety of CE-224,535, an antagonist of P2X7 receptor, in treatment of patients with rheumatoid arthritis inadequately controlled by methotrexate. J Rheumatol. 2012;39:720–7. 22382341 10.3899/jrheum.110874

[79] Lau OC, Samarawickrama C, Skalicky SE. P2Y2 receptor agonists for the treatment of dry eye disease: a review. Clin Ophthalmol. 2014;8:327–34. 24511227 10.2147/OPTH.S39699 PMC3915022

[80] Shahidullah M, Wilson WS. Mobilisation of intracellular calcium by P2Y2 receptors in cultured, non-transformed bovine ciliary epithelial cells. Curr Eye Res. 1997;16:1006–16. 9330852 10.1076/ceyr.16.10.1006.9018

[81] Boucher I, Rich C, Lee A, Marcincin M, Trinkaus-Randall V. The P2Y2 receptor mediates the epithelial injury response and cell migration. Am J Physiol Cell Physiol. 2010;299:C411–21. 20427708 10.1152/ajpcell.00100.2009 PMC2928627

[82] Rius-Pérez S, Pérez S, Toledano MB, Sastre J. Mitochondrial reactive oxygen species and lytic programmed cell death in acute inflammation. Antioxid Redox Signal. 2023;39:708–27. 37450339 10.1089/ars.2022.0209 PMC10619893

[83] Hale LP, Braun RD, Gwinn WM, Greer PK, Dewhirst MW. Hypoxia in the thymus: role of oxygen tension in thymocyte survival. Am J Physiol Heart Circ Physiol. 2002;282:H1467–77. 11893584 10.1152/ajpheart.00682.2001

[84] Braun RD, Lanzen JL, Snyder SA, Dewhirst MW. Comparison of tumor and normal tissue oxygen tension measurements using OxyLite or microelectrodes in rodents. Am J Physiol Heart Circ Physiol. 2001;280:H2533–44. 11356608 10.1152/ajpheart.2001.280.6.H2533

[85] Minutoli L, Puzzolo D, Rinaldi M, Irrera N, Marini H, Arcoraci V, et al. ROS-mediated NLRP3 inflammasome activation in brain, heart, kidney, and testis ischemia/reperfusion injury. Oxid Med Cell Longev. 2016;2016:2183026. 27127546 10.1155/2016/2183026 PMC4835650

[86] Pétrilli V, Papin S, Dostert C, Mayor A, Martinon F, Tschopp J. Activation of the NALP3 inflammasome is triggered by low intracellular potassium concentration. Cell Death Differ. 2007;14:1583–9. 17599094 10.1038/sj.cdd.4402195

[87] Tschopp J, Schroder K. NLRP3 inflammasome activation: the convergence of multiple signalling pathways on ROS production? Nat Rev Immunol. 2010;10:210–5. 20168318 10.1038/nri2725

[88] Heid ME, Keyel PA, Kamga C, Shiva S, Watkins SC, Salter RD. Mitochondrial reactive oxygen species induces NLRP3-dependent lysosomal damage and inflammasome activation. J Immunol. 2013;191:5230–8. 24089192 10.4049/jimmunol.1301490 PMC3833073

[89] Pernes G, Flynn MC, Lancaster GI, Murphy AJ. Fat for fuel: lipid metabolism in haematopoiesis. Clin Transl Immunology. 2019;8:e1098. 31890207 10.1002/cti2.1098 PMC6928762

