# Supplementary material for: Damage-induced pyroptosis drives endogenous thymic regeneration by activating the purinergic receptor P2Y2
Source: Cell Death Dis. 2026 Jan 3;17(1):157. doi: 10.1038/s41419-025-08345-x (PMC12859001; doi:10.1038/s41419-025-08345-x)
Supplement: Supplementary file 1 — Supplementary Figure 1 [file 41419_2025_8345_MOESM1_ESM.pdf]

Figure S1

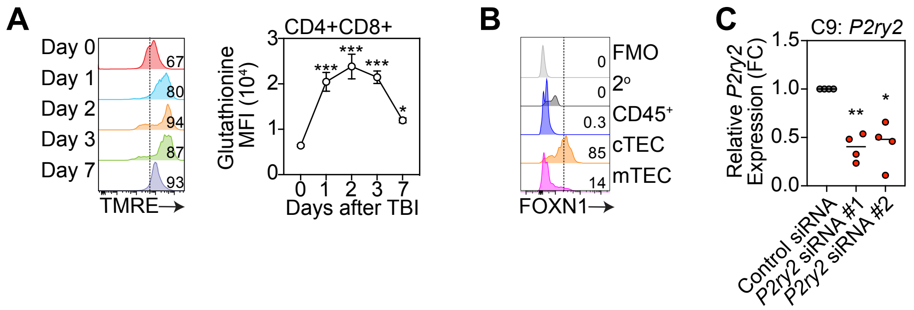

Supplementary Figure 1: A, Thymuses from 6–8-week-old C57BL/6 mice were harvested at days 0, 1, 2, 3 and 7 following TBI and glutathione levels were measured by flow cytometry in DP thymocytes (n=3 mice)**; B,** Steady-state thymuses were harvested from 8-week-old C57BL/6J mice and digested enzymatically. The cells were plated at 2 x 10^6 cells/well and stimulated with 100 μM or 300μM bzATP. Histograms of FOXN1+ cTECs, mTECs and CD45+ cells; **C,** qPCR of Foxn1 expression in C9s at day 3 following siRNA transfection (n=4 independent experiments, two-tailed t test).
