# Supplementary material for: Damage-induced pyroptosis drives endogenous thymic regeneration by activating the purinergic receptor P2Y2
Source: Cell Death Dis. 2026 Jan 3;17(1):157. doi: 10.1038/s41419-025-08345-x (PMC12859001; doi:10.1038/s41419-025-08345-x)

Caspase 1 Western Blot

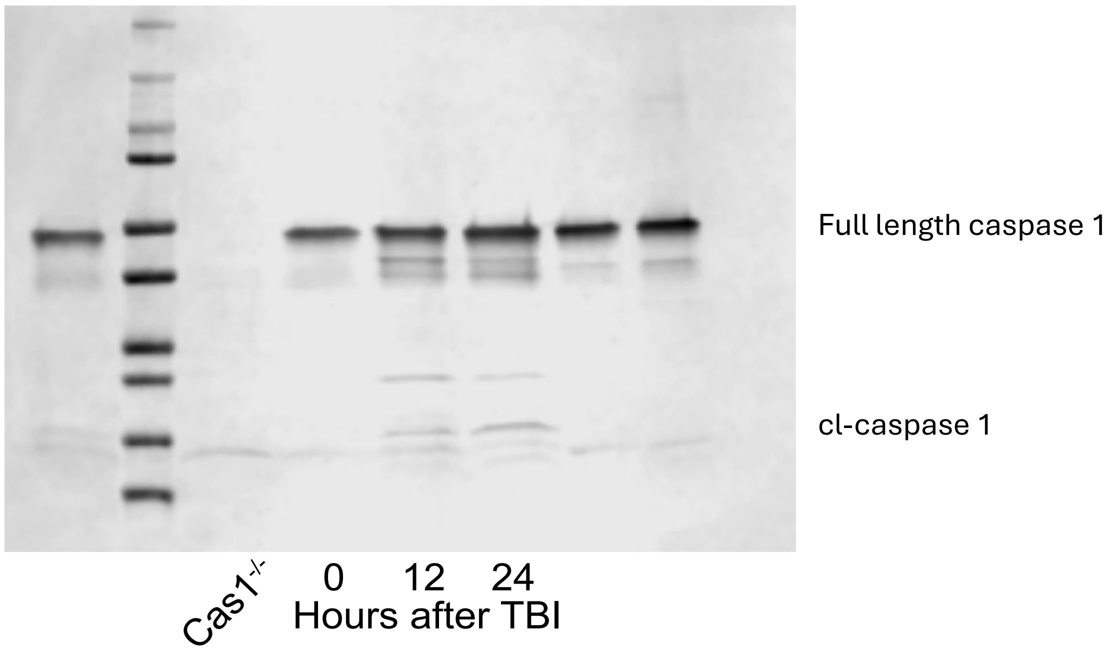

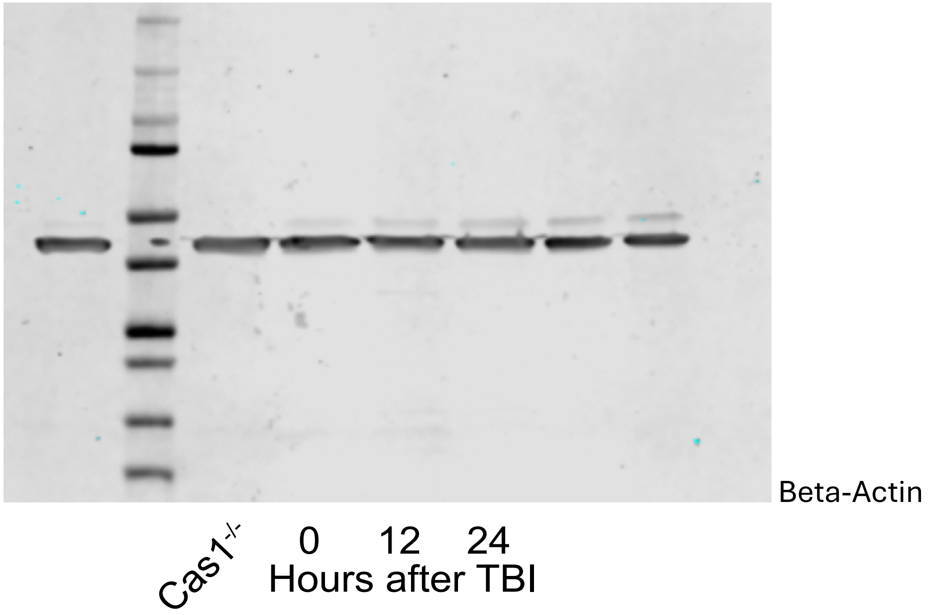

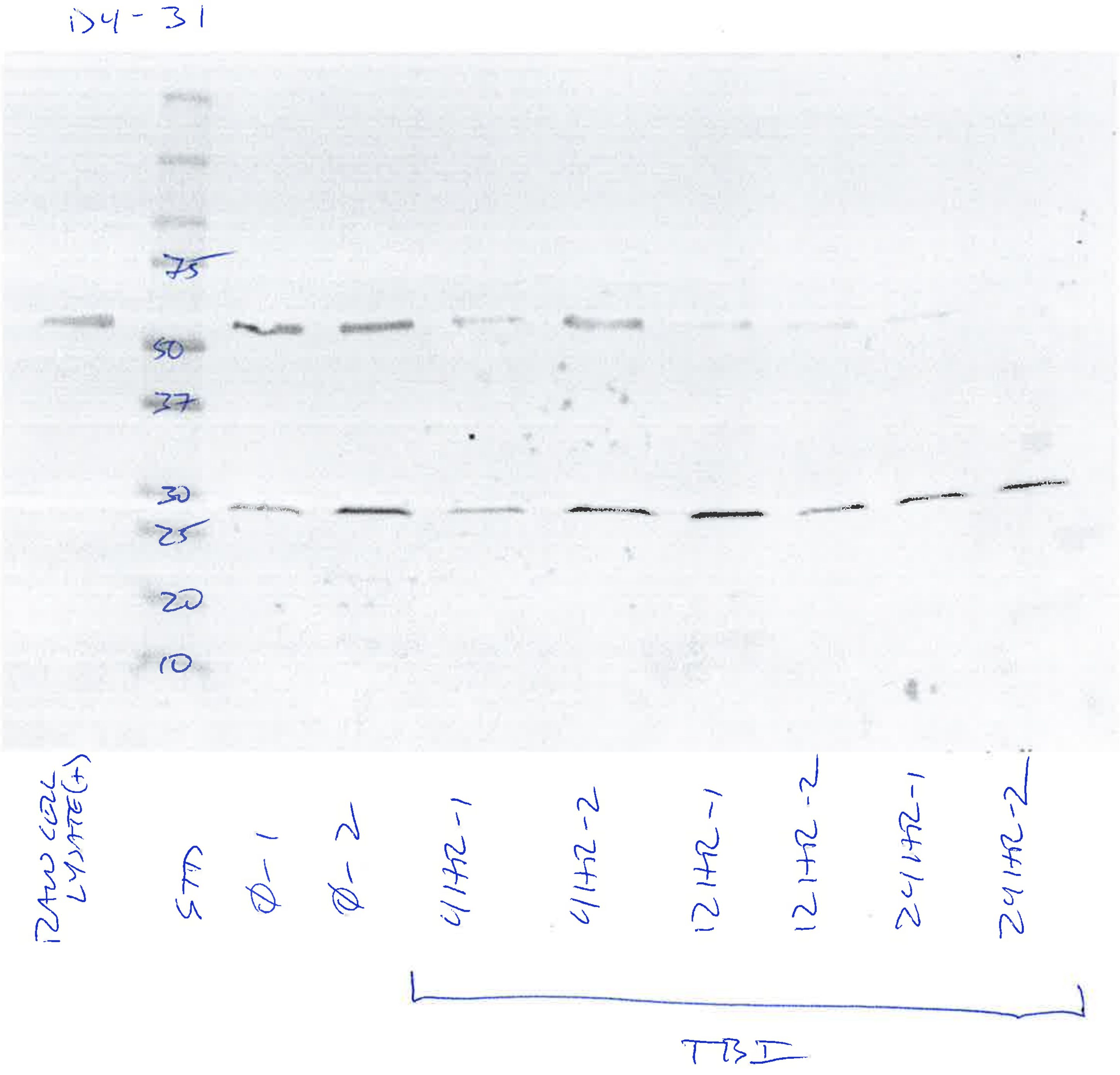

D4-31
75
50
37
30
25
20
10
RAW CELL LYSATE(+)
STD
Ø-1
Ø-2
6HR-1
6HR-2
12HR-1
12HR-2
24HR-1
24HR-2
TBI

D4-25 α GASDERMIN D

FULL LENGTH = 53 KD
CLEAVED = 30 KD

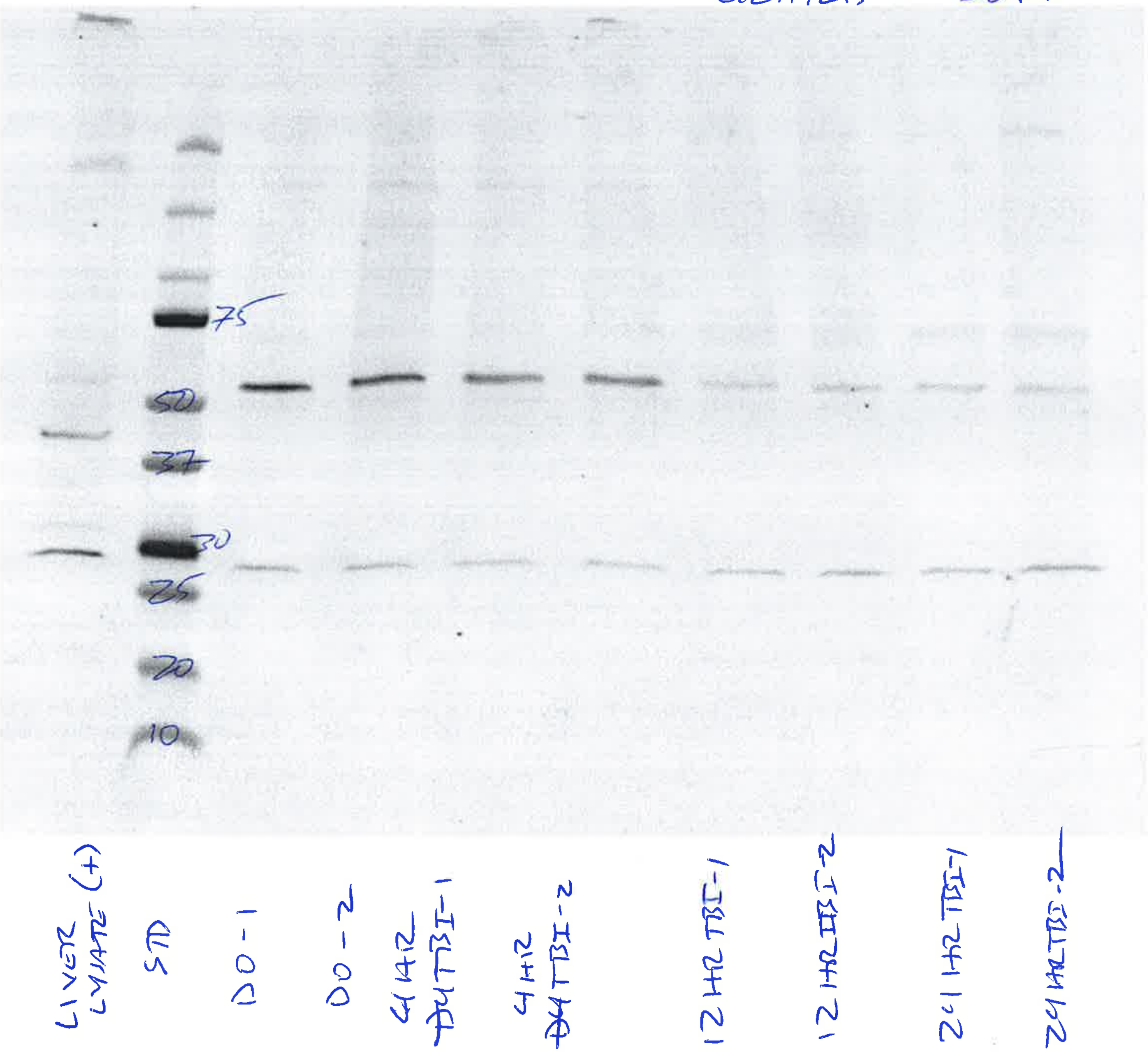

Supplement: Supplementary file 2 — Uncropped Western Blots [file 41419_2025_8345_MOESM2_ESM.pdf]
